# Fractional flow reserve-guided management in stable coronary disease and acute myocardial infarction: recent developments

**DOI:** 10.1093/eurheartj/ehv206

**Published:** 2015-06-02

**Authors:** Colin Berry, David Corcoran, Barry Hennigan, Stuart Watkins, Jamie Layland, Keith G. Oldroyd

**Affiliations:** 1 West of Scotland Heart and Lung Centre, Golden Jubilee National Hospital, Clydebank, UK; 2 BHF Glasgow Cardiovascular Research Centre, Institute of Cardiovascular and Medical Sciences, University of Glasgow, 126 University Place, Glasgow G12 8TA, UK; 3 St Vincent's Hospital, Melbourne, Australia

**Keywords:** Fractional flow reserve, Stable angina, Myocardial infarction, Medical therapy, Coronary revascularization

## Abstract

Coronary artery disease (CAD) is a leading global cause of morbidity and mortality, and improvements in the diagnosis and treatment of CAD can reduce the health and economic burden of this condition. Fractional flow reserve (FFR) is an evidence-based diagnostic test of the physiological significance of a coronary artery stenosis. Fractional flow reserve is a pressure-derived index of the maximal achievable myocardial blood flow in the presence of an epicardial coronary stenosis as a ratio to maximum achievable flow if that artery were normal. When compared with standard angiography-guided management, FFR disclosure is impactful on the decision for revascularization and clinical outcomes. In this article, we review recent developments with FFR in patients with stable CAD and recent myocardial infarction. Specifically, we review novel developments in our understanding of CAD pathophysiology, diagnostic applications, prognostic studies, clinical trials, and clinical guidelines.

## Introduction

Fractional flow reserve (FFR) is a whole cardiac cycle pressure-derived index of the maximum achievable blood flow in a coronary artery with a stenosis expressed as a ratio of maximum achievable blood flow if that artery were normal.^1^ Fractional flow reserve is a means of assessing the physiological significance of a coronary artery stenosis. Some of the most important clinical trials involving patients with coronary artery disease (CAD) have assessed and confirmed the validity of FFR as a predictor of outcome. Fractional flow reserve citations in biomedical journals are increasing^2^ and FFR-guided management in patients with stable CAD now has Class I and Class IIa guideline recommendations.^3,4^

We consider recent studies clarifying further the role of FFR in patients with stable CAD and myocardial infarction (MI). We focus on research published from 2013 to the present, whilst also citing relevant landmark publications. We have searched databases, i.e. PUBMED and registries, i.e. http://www.clinicaltrials.gov, using the following key words: ‘fractional flow reserve’, ‘coronary physiology’, ‘diagnostic’, ‘stable coronary disease’, ‘acute coronary syndrome’, ‘myocardial infarction’, ‘observational study’, and ‘clinical trial’. The results include an assessment of study quality criteria^5,6^ (Supplementary material online, *File S1*).

## Fractional flow reserve: new insights into clinical significance

Coronary artery disease is a leading global cause of morbidity and mortality.^7–9^ Invasive angiography is the reference test for the diagnosis of CAD. However, the relationship between angiographic stenosis severity and coronary blood flow is complex. Visual assessment of stenosis severity is subjective and correlates poorly with physiological significance. More objective measurements of stenosis severity using quantitative coronary analysis (QCA) are also commonly discordant with the physiological significance of the lesion, as defined by FFR (≤0.80).^10^

## Coronary physiology and stenosis flow dynamics

In the presence of an obstructive epicardial coronary stenosis, perfusion pressure is reduced leading to compensatory vasodilatation,^1,11,12^ which itself has prognostic importance.^13^ The total pressure drop across a stenosis is defined by the sum of the viscous friction along the entrance of a lesion (increasing with flow in a linear manner, Poiseuille's Law), and losses incurred by convective acceleration along the lesion (increasing with the square of flow, Bernoulli's Law). Due to flow separation and eddy formation, these pressure losses are not recovered at the stenosis exit.

## Fractional flow reserve

**Figure 1:**
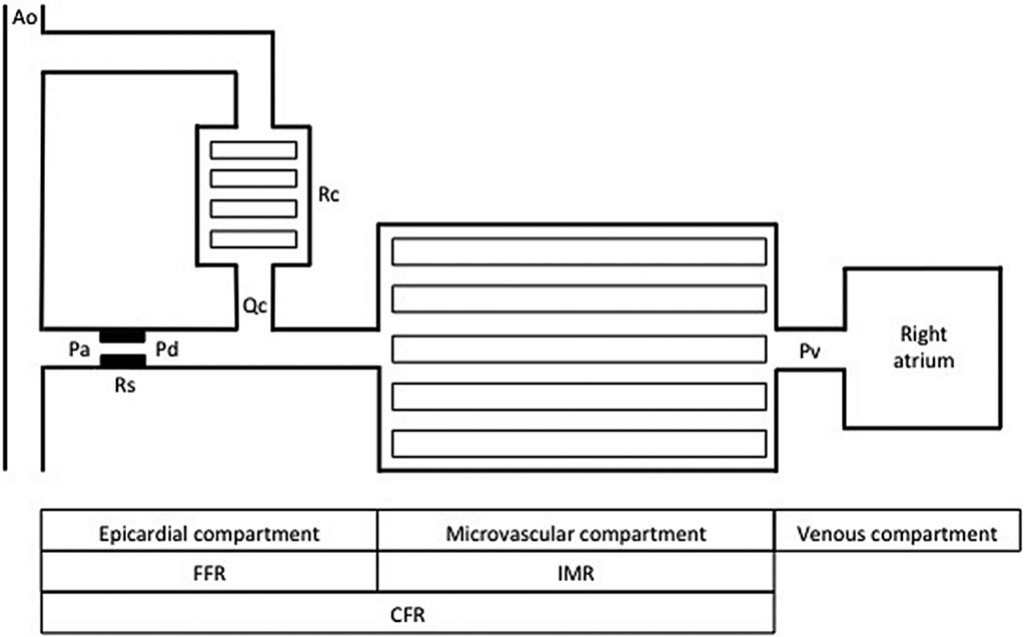
Systemic and coronary vascular beds that influence FFR.^14^ Ao, aortic pressure; Pa, arterial pressure proximal to stenosis; *P*_d_, coronary pressure distal to epicardial stenosis; *P*_v_, venous pressure; *Q*_c_, collateral blood flow; *R*_c_, collateral resistance; *R*_s_, epicardial coronary stenosis; FFR, fractional flow reserve; IMR, index of microvascular resistance; CFR, coronary flow reserve.

When coronary resistance is minimized by pharmacological vasodilatation, there is an approximately linear (more accurately defined as incremental-linear) relationship between perfusion pressure and blood flow within the physiological blood pressure range.^1,11^ Myocardial FFR (FFRmyo) is defined as the maximal blood flow to the subtended myocardium in the presence of a stenosis compared with maximal flow in the absence of a stenosis.^1,14^ Using a pressure-sensitive coronary wire, FFR is calculated from the coronary pressure distal to a stenosis (*P*_d_) and the aortic pressure (*P*_a_) obtained simultaneously by a haemodynamic pressure transducer, both measured under conditions of maximal hyperaemia: FFRmyo = *P*_d_/*P*_a_ (*Figure 1*).^14^

By measuring the coronary wedge pressure (*P*_w_) during maximum hyperaemia, the contribution to myocardial blood flow from sources other than the epicardial artery (such as collateral and venous flow) can be assessed and analysed separately.^14^ This is the coronary FFR (FFRcor = [*P*_d_− *P*_w_]/[*P*_a_− *P*_w_]). Myocardial FFR is normally used in practice and in most cases incorporation of *P*_w_ makes no difference to the decision for revascularization.^14^

## Fractional flow reserve threshold for ischaemia

Based on repeated non-invasive stress testing, the FFR threshold for discriminating clinically significant lesion-level ischaemia is 0.75,^15^ and revascularization decisions based on this threshold are evidence-based.^16^ In order to increase measurement sensitivity to reliably exclude the presence of functionally significant stenoses, a threshold of 0.80 has been adopted and is now evidence-based.^17–19^ However, when a treatment decision is made involving FFR, it is good practice to take account of other clinical information, including the medical history, CAD characteristics, and the myocardial territory-at risk.^3,4^

### When should central venous pressure be measured?

Strictly, the calculation for FFR should account for right atrial pressure (*P*_v_): FFRmyo = [*P*_d_ − *P*_v_]/[*P*_a_ − *P*_v_].^14^ However, pragmatically, venous pressure is not usually measured in daily clinical practice and *P*_v_ was not used for FFR calculation in the landmark clinical trials.^17–19^ This reflects the fact that Pv has minimal influence on FFR or revascularization decisions. In general, paradoxical vasoconstriction of the coronary microcirculation does not occur even in the presence of a severe stenosis.

## Pharmacological hyperaemia

Induction of maximal vasodilatation through reductions in myocardial and collateral circulatory resistances is required for accurate measurement of FFR.^1,14^ The standard approach for FFR measurement involves administration of intravenous adenosine at a dose of 140 µg/kg/min.^20^ Intravenous adenosine reduces systemic and coronary vascular resistance and these changes are correlated.^21^

### Fractional flow reserve reproducibility

The VERification of Instantaneous wave-Free ratio and fractional flow reserve for the assessment of coronary artery stenosis severity in everydaY practice (VERIFY) was a prospective study of 206 consecutive patients with an indication for an FFR measurement who were simultaneously enrolled in six European centres (4 January–10 February 2012).^22^ Fractional flow reserve was measured using 140 μg/kg/min of intravenous adenosine administered for 2 min and then again after a 2 min rest period. Fractional flow reserve data were assessed by a central laboratory. The FFR reproducibility was high (*r*^2^ = 0.98) and the limits of agreement were narrow (−0.04 to −0.04). Other studies have also shown minimal FFR variation with different doses of intravenous adenosine.^23,24^

### Factors that influence the response to pharmacological vasodilatation and fractional flow reserve

Treatment decisions should be based on the minimum FFR value^1,14,15^ which typically occurs shortly after the onset of steady-state hyperaemia.^25^ Occasionally, the minimum FFR value may occur slightly before steady-state hyperaemia, implying that the *P*_d_/*P*_a_ ratio may not equal the maximum coronary flow ratio between stenosed and normal artery.^26^ In this case, the steady-state FFR may be slightly higher than the minimum FFR in which case the steady-state FFR should be considered for decision-making.^26^

Fractional flow reserve values based on at least a 3-beat average should minimize beat-to-beat variability.^25^ Fractional flow reserve measurements across serial lesions should be done slowly in order to reveal the minimum FFR at specific locations and repeated measurement of FFR would seem good practice.

Matsuomo *et al*.^27^ studied the influence of caffeine (an adenosine receptor antagonist) on FFR. They observed that in 28 patients with detectable blood caffeine concentrations FFR values increased with incremental doses of adenosine (140, 175, and 210 µg/kg/min) compared with intra-coronary papaverine (10–20 mg) whereas the FFR results were unrelated to the dose of adenosine in 14 patients who had avoided caffeine for >24 h.

### Route of adenosine administration

For intravenous adenosine, steady-state hyperaemia typically requires at least 1 min to develop meaning that the cardiologist should allow sufficient time for steady-state conditions to be established.^25,26,28^ Lim *et al*.^23^ studied a cohort of patients who had an FFR evaluation with intravenous and intra-coronary adenosine in 238 lesions. They found a high degree of classification agreement (92.9%; Cohen's kappa = 0.887) for the intravenous vs. intra-coronary routes of administration. Seto *et al*.^25^ found that peripheral and central venous routes of adenosine administration were associated with similar minimum FFR values.

Intra-coronary adenosine is sometimes preferred in practice as it is simpler to administer and potentially less expensive. In a recent dose–response study of intra-coronary adenosine on coronary flow velocity, Adjedj *et al*.^29^ found that 60–100 µg of adenosine in the right coronary artery and 160–200 µg of adenosine in the left coronary artery safely induced maximum hyperaemia. The Can cONTrast Injection Better Approximate FFR compAred to Pure reSTing Physiology (CONTRAST; NCT02184117) study is a prospective multicentre study involving 750 patients with a clinical indication for FFR.^30^ A pre-specified aim of this study is to assess the equivalence between FFR measured using intra-coronary and intravenous adenosine. Given the prospective design and large sample size, this study could provide conclusive information on the utility of intra-coronary adenosine for pharmacological hyperaemia.

### Non-adenosine methods of pharmacological vasodilatation

Vasodilators other than adenosine have been assessed for FFR measurement, including intravenous regadenoson (a specific A2A receptor agonist),^24^ and intra-coronary sodium nitroprusside,^31^ nicorandil,^32^ nitrate,^33^ and papaverine.^34^ Fractional flow reserve responses are broadly comparable for these different pharmacological vasodilator agents.^23^

## Stenosis morphology and functional significance

### Coronary artery stenosis characteristics and fractional flow reserve

The FFR and Intravascular Ultrasound Relationship Study (FIRST) was a prospective, multicentre, international registry of 350 patients [367 lesions; 55% stable angina, 42% acute coronary syndrome (ACS)] that assessed the relationships between coronary lesion characteristics revealed by intravascular ultrasound (IVUS) and FFR.^34^ A minimum lumen area (MLA) of <3.07 mm^2^ had moderate accuracy [area-under-the curve (AUC) = 0.65] for identifying an FFR of <0.80, and the AUC increased with increasing vessel diameter (a surrogate for subtended myocardial volume). Plaque burden had a weak positive correlation with FFR (*r* = −0.22, *P* < 0.001). Thin-cap fibroatheroma and calcification were associated with lower correlations between MLA and FFR. The multivariable correlates of FFR were MLA by IVUS, diameter stenosis by QCA, and left anterior descending (LAD) coronary artery (vs. right coronary artery).

Iguchi *et al*.^35^ found a strong inverse correlation between lesion length and FFR value. López-Palop *et al*.^36^ suggested that a length of >20 mm was the strongest morphological determinant of functional significance. Takashima *et al*.^37^ found that lesion complexity (assessed by QCA) correlated with FFR, with the hypothesis that with increasing complexity there are greater pressure losses due to flow separation and friction. In a multivariate analysis, Cho *et al*.^38^ assessed the factors associated with mismatch between FFR and QCA in 643 lesions (*n* = 462 patients). They observed that lesion location (LAD vs. non-LAD) predicted FFR and that QCA parameters were more likely to over-estimate stenosis severity (vs. FFR) in non-LAD lesions and short lesions. Reference vessel diameter and multi-vessel disease were associated with over- and underestimation of the physiological significance, respectively. Leone *et al*.^39^ found an inverse correlation between the amount of subtended myocardium and the FFR value. Quantitative coronary analysis cannot accurately predict FFR as it does not account for the volume of distribution of the coronary artery nor for the function of the microcirculation within that coronary territory. In summary, morphological parameters of stenosis severity influence FFR but are not a reliable substitute for physiological assessment.

### Age and gender influences on fractional flow reserve

Fractional flow reserve measured in a coronary artery without atherosclerosis approximates 1.0 irrespective of age or sex, whereas this may not be the case in flow-derived indices such as coronary flow reserve (CFR) or hyperaemic stenosis resistance (HSR). Age-related changes in cardiac structure (e.g. interstitial fibrosis) and coronary disease (e.g. microvascular dysfunction) may influence FFR. In a *post hoc* analysis of the FAME trial participants, Lim *et al*.^40^ observed that the mean FFR value obtained in older patients (>65 years) was higher than that in patients <65 years (0.72 ± 0.17 vs. 0.70 ± 0.18; *P* = 0.043), and that for any given angiographic stenosis severity the FFR value was more likely to be higher in older subjects. However, FFR-guided percutaneous coronary intervention (PCI) was equally beneficial regardless of age,^40^ reflecting similar potential for flow-augmentation after PCI.^1,11,12^ Ageing was also a multivariable associate of overestimation of disease severity in the analysis by Cho *et al.*^38^

The IRIS FFR-DEFER registry of 700 patients reported that for the same degree of angiographic stenosis severity women were more likely to have higher FFR values.^41^ Li *et al*.^42^ reported a similar finding in a retrospective study of 1090 patients. The potential explanations for this discrepancy include an increased prevalence of microvascular disease in females and a lower body surface area and myocardial mass resulting in a smaller subtended myocardial territory for a given stenosis compared with males.

### Fractional flow reserve and microvascular function: complementary use of fractional flow reserve, coronary flow reserve, and index of microvascular resistance

The index of microvascular resistance (IMR) is a guidewire-based quantitative measure of microvascular resistance.^43–46^ As IMR is measured during hyperaemia, it is less dependent on haemodynamic variations and has better repeatability than CFR. Index of microvascular resistance is calculated from distal coronary pressure (*P*_d_) multiplied by the mean transit time (*T*_mn_) of a 3 mL bolus of room temperature saline during hyperaemia induced by intravenous adenosine, where IMR = *P*_d_ × *T*_mn_. An IMR < 25 is considered normal, with values greater than this consistent with microvascular dysfunction.

#### Complementary use of fractional flow reserve, coronary flow reserve, and index of microvascular resistance in daily clinical practice

**Figure 2:**
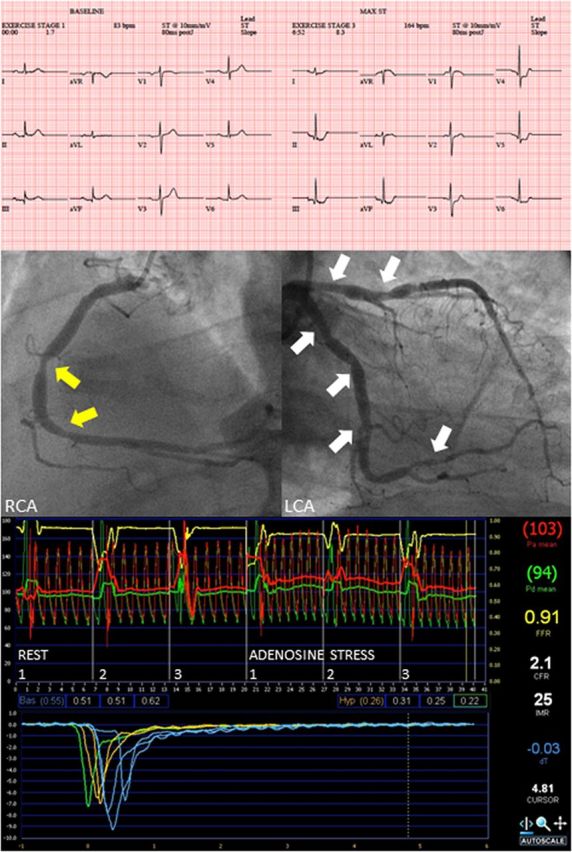
Angina associated with inducible ischaemia, non-obstructive epicardial coronary artery disease and microvascular dysfunction revealed by guidewire-based diagnostic tests with FFR, CFR, and IMR. A 70-year-old male presented to the Chest Pain Service with a 2-month history consistent with Canadian Cardiovascular Society class II angina and hypertension. A treadmill exercise tolerance test disclosed angina and ST-segment depression in leads II, III, aVF, and V3–V6 at 6 min 52 s at Stage 3 of the Bruce protocol. The patient was invited to participate in the CE-MARC2 clinical trial (NCT01664858).^94^ Written informed consent was obtained and he was then randomly assigned to the management according to the National Institute of Clinical Excellence guideline-95. Based on a pre-test likelihood of coronary artery disease of 60–90%, the patient was referred directly for invasive management. Coronary angiography revealed multiple plaques (white arrows) in the left (white arrow) and right (yellow arrow) coronary arteries. The FFR in all three major epicardial arteries was >0.90, ruling out flow-limiting stenoses in these arteries. The IMR and CFR were 31 and 1.4 in the left circumflex artery and 25 and 2.1 in the right coronary artery, consistent with microvascular dysfunction. The haemodynamic display from guidewire-based physiological testing in the right coronary artery shows recordings of pressure (upper panel) measured from the guide catheter in the aorta (red, Ao) and guidewire-based distal coronary pressure (green, *P*_d_), and thermodilution curves (lower panel) using serial intra-coronary injections of 3 mL of saline at room temperature at rest (blue thermodilution curves, three curves, mean 0.55 s) and then during adenosine stress [orange thermodilution curves with one highlighted in green (active measurement, 0.22 s, mean 0.26 s) during a 41 s measurement period (*x*-axis)]. There is a modest ‘left-shift’ in the thermodilution transit times indicating a reduced vasodilator response of the coronary microcirculation to intravenous adenosine, consistent with a degree of microvascular dysfunction. The patient was treated medically with 75 mg of aspirin, 40 mg of simvastatin, and angina medications. Permission obtained, Prof. John Greenwood, Principal Investigator for the CE-MARC2 trial.^94^ RCA, right coronary artery; LCA, left coronary artery; FFR, fractional flow reserve; CFR, coronary flow reserve; IMR, index of microcirculatory resistance.

Combining FFR with CFR and IMR measurements in daily clinical practice can give clinicians instantaneous and complementary diagnostic information on epicardial CAD and microvascular function in the catheter laboratory (for reviews, 46–48; *Figure 2*). This approach may be particularly relevant in a subset of patients presenting with angina, non-invasive evidence of ischaemia, but no significant epicardial CAD (FFR > 0.80).^49,50^ In patients with non-obstructive atheroma (FFR > 0.8), an impaired CFR and an increased IMR indicates the presence of coronary microvascular disease. In contrast, in patients without obstructive epicardial CAD (by angiography or FFR), an impaired CFR and a normal IMR, diffuse atherosclerotic CAD may cause ‘low-flow’ ischaemia.^47^ In this situation, increasing atherosclerotic plaque burden is offset by vessel remodelling which preserves the vessel lumen diameter. With no focal stenosis, there is a lack of convective acceleration through the vessel and thus distal pressure loss is limited (FFR > 0.8) even if CFR is significantly impaired.^48^ The discordance between FFR and CFR (i.e. normal FFR and abnormal CFR) may be prognostically important.^51^ The Distal Evaluation of Functional Performance With Intravascular Sensors to Assess the Narrowing Effect – Combined Pressure and Doppler FLOW Velocity Measurements (DEFINE-FLOW) study will prospectively examine a management strategy that combines FFR and CFR to inform treatment decisions in 500 patients with CAD. In this study, patients with a reduced FFR but preserved CFR (>2.0) will be treated medically and PCI will only be performed when FFR and CFR are reduced.^52^

#### Other physiological indices

Hyperaemic stenosis resistance is derived from coronary pressure and Doppler flow measurements.^53^ The validity of HSR has been prospectively assessed in a single head-to-head comparison with myocardial perfusion scintigraphy with Myoview involving doses of intra-coronary adenosine (20–40 µg) that may have induced sub-maximal hyperaemia.^53^ Further validation of HSR has been undertaken involving *post hoc* ROC analyses.^54^ Wider adoption of Doppler-based indices is limited by reproducibility^55^ and pressure-flow diagnostic wires are more expensive than pressure-only wires.

## Recent developments with fractional flow reserve

### Contrast fractional flow reserve

Radiographic contrast media have vasodilator properties and, if *P*_d_/*P*_a_ measured during contrast-induced hyperaemia is already <0.80, then pharmacological vasodilation might be obviated. Leone *et al*.^56^ assessed 104 coronary stenoses in 80 consecutive patients. They found that *P*_d_/*P*_a_ derived from contrast media was slightly higher than FFR derived from intravenous adenosine. The correlation between these two parameters was strong (*r* = 0.94, *P* < 0.001) and at a cut-off of ≤0.83 contrast *P*_d_/*P*_a_ had high diagnostic accuracy for FFR ≤ 0.80 [AUC = 0.97 (95% CI 0.91–0.99, specificity = 96.1%, sensitivity = 85.7%)]. The primary outcome of the CONTRAST study^30^ is the improvement in agreement from resting indexes (rest *P*_d_/*P*_a_ and iFR™) to contrast *P*_d_/*P*_a_, using FFR ≤ 0.8 as the binary reference standard.

### Smart minimum fractional flow reserve

During a continuous recording with the pressure sensor at a fixed position in a coronary artery *P*_d_/*P*_a_ may fluctuate. Focusing on the inherent variability in coronary pressure recordings, Johnson *et al.*^57^ demonstrated that despite fluctuating haemodynamics the minimum measured FFR value is highly repeatable. They have developed a novel ‘smart minimum’ algorithm to select out the highest quality FFR data within a recording, which should help cardiologists to identify the minimum FFR value for decision-making. The algorithm is generic and not commercially restricted.

### Diastolic fractional flow reserve

Coronary blood flow is predominantly in diastole and segmentation of FFR to diastole (dFFR) might have higher diagnostic accuracy for the detection of ischaemia.^58,59^ In a *post hoc* analysis of the VERIFY study, we found near equivalent diagnostic accuracy for whole-cycle and dFFR with an AUC of 98% in an ROC analysis for diastolic FFR predicting FFR ≤ 0.80 (N.L. Johnson, personal communication). In reality, full-cycle FFR and dFFR have similar diagnostic value.

## Fractional flow reserve estimated from cardiac imaging based on computational fluid dynamics

### Fractional flow reserve from computed tomography coronary angiography

Fractional flow reserve can now be estimated non-invasively from high-quality computed tomography (CT) coronary angiograms. The DISCOVER-FLOW study examined the relationships between non-invasive FFR (FFR-CT) vs. FFR measured invasively in 159 arteries in 103 patients and reported that adoption of FFR-CT markedly improved the diagnostic accuracy of the CT scan.^60^ The larger HEARTFLOW-NXT trial incorporated further developments with the FFR-CT technology and in a stringent protocol involving a selected patient population diagnostic accuracy for FFR-CT was further improved.^61^

### Fractional flow reserve from invasive coronary angiography

Fractional flow reserve can be estimated using 3-D angiography, TIMI frame count (FFR-QCA),^62^ and also from rotational angiography images alone [virtual FFR (vFFR)^63^]. These promising developments should undergo further studies in larger unselected patient populations.

## Resting pressure indices measured invasively: an alternative to fractional flow reserve?

Mamas *et al*.^64^ originally described the relationships between resting and hyperaemic pressure measurements in 528 pressure wire recordings obtained in 483 patients. They found that for an FFR ischaemic threshold (≤0.75), a whole-cycle *P*_d_/*P*_a_ cut-off of ≤0.85 had a positive predictive value of 95% and *P*_d_/*P*_a_ of ≥0.93 had a negative predictive value of 95.7%. One other study reported near 100% diagnostic accuracy with a *P*_d_/*P*_a_ adenosine zone of 0.87–0.99.^65^

### The instantaneous wave-free ratio (iFR™)

iFR™ involves estimation of the trans-stenotic pressure gradient at rest during a time interval starting 25% into diastole and ending 5 ms before the onset of systole using a trademarked algorithm. iFR™ was originally described in 2011 by the ADVISE investigators who proposed that an iFR™ cut-off value of 0.83 was equivalent to an FFR value of 0.80.^66^ More recently this cut-off value has been revised upwards to 0.89–0.90^67^ and a hybrid strategy is also proposed.^68^ The VERIFY^22^ and RESOLVE^69^ studies called into question the diagnostic accuracy of resting pressure indices vs. FFR stimulating further investigations,^30,67–75^ including head-to-head comparisons of iFR™ vs. FFR-guided management in clinical trials designed to assess health outcomes.^74,75^

## Fractional flow reserve in stable coronary disease: results from single and multicentre cohort studies

Park *et al*.^76^ reported data from the large single-centre ASAN PCI registry in which 2699 patients had PCI performed before FFR was in routine use and 2398 after it became the standard of care. Fractional flow reserve was used in lesions with diameter stenosis severity between 50 and 80% when there was no prior evidence of ischaemia. Percutaneous coronary intervention was undertaken if the FFR is <0.75 and deferred if FFR is >0.8. ‘Grey zone’ results were left to the operator's discretion. The primary endpoint of the study was a combination of death from any cause, MI, and repeat revascularization at 1 year. In a propensity-matched analysis, compared with the angiography-guided population, the primary endpoint was lower in the FFR-guided population (8.6 vs. 4.8%, *P* < 0.001) with a hazard ratio of 0.55 (95% CI 0.43–0.7, *P* < 0.001). This result was mainly attributed to less peri-procedural MI and repeat revascularization, despite a lower use of stents.

Frohlich *et al*.^77^ analysed the London PCI registry (2004–2011) in which FFR was used according to operator discretion. The FFR group had an unadjusted all-cause mortality benefit (HR 0.72, 95% CI 0.61–0.84, *P* < 0.001) though there was no association with mortality when the data were adjusted for confounders. A propensity-matched analysis of FFR vs. angiography-alone (919 pairs) showed no difference in mortality (*P* = 0.32) but with a lower mean stent number in the FFR group (1.1 vs. 1.7, *P* < 0.001). The publication lacked information on medically managed patients.

Li *et al*.^78^ reported follow-up results of a similar analysis of 7050 patients from the Mayo Clinic registry (2002–2009). They found lower MACE with FFR guidance compared with angiographic guidance (50 vs. 57%, *P* = 0.016). Fractional flow reserve-guided revascularization in patients with bypass grafts is also associated with a better outcome and lower costs.^79^

### Deferral of revascularization and adoption of medical therapy alone

Depta *et al*.^80,81^ retrospectively analysed the outcomes of 720 (881 intermediate lesions) patients in whom PCI was deferred following FFR measurement between 2002 and 2010. Lesions were divided into three groups according to FFR: grey zone (0.75–0.8; *n* = 65); borderline (0.81–0.85; *n* = 275), and non-borderline (>0.85; *n* = 541). One hundred and fifty-seven (18%) patients required deferred lesion intervention during 4.5 ± 2.1 years follow-up. Of these, 117 had PCI and 40 had coronary artery bypass graft surgery (CABG). Overall, one in four stenoses with a borderline FFR (0.81–0.85) required intervention during the study follow-up period. The rate of subsequent MI following deferral based on FFR was 11% and the deferred lesion was the culprit in 38%.

Van de Hoef *et al*.^13^ measured FFR and coronary flow velocity reserve (CFVR) in one intermediate stenosis in 157 patients in whom revascularization was deferred. During 10 years of follow-up, a normal FFR with abnormal CFVR was associated with more MACE regardless of whether an FFR cut-off of 0.75 or 0.80 was used. One explanation for this discordance is that whilst successful revascularization should negate the prognostic impact of a lesion with an ischaemic FFR, this effect may be attenuated if post-PCI CFR remains abnormal. The DEFINE-FLOW study will focus on this question.^52^

### Reclassification of treatment decisions during angiography

The RIPCORD study was designed to assess whether routine FFR measurement during diagnostic coronary angiography would impact the management of patients when compared with angiographic assessment.^82^ Two hundred patients with stable angina were enrolled in 10 UK centres. The main result was that the management plan (medical therapy alone, PCI, CABG, or more information required) changed in 26% of the population. The results of the Registre Français de la FFR (R3F) in 1075 consecutive patients enrolled in 20 centres^83^ were consistent with those of RIPCORD in that reclassification of management with FFR was common (43% of the cases; revascularization reduced overall). In addition, clinical outcomes at 1 year were not compromised by deciding the treatment plan based on FFR.

### Prognostic importance of fractional flow reserve

**Figure 3:**
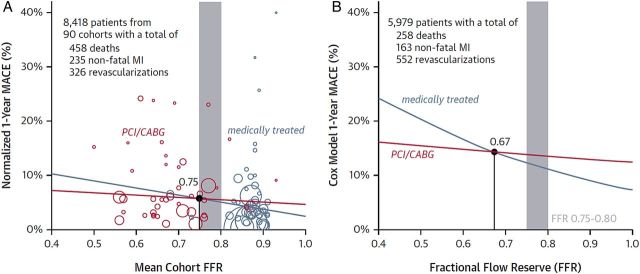
Prognostic importance of fractional flow reserve values below and above the ischaemic zone (0.75–0.80).^84^

The meta-analysis of study-level (*n* = 9173) and individual patient-level (*n* = 6961) data by Johnson *et al*.^84^ has provided new information on the prognostic importance of individual FFR values. Put simply, clinical events increased as FFR decreased and FFR measured post-PCI had an inverse relationship with prognosis (hazard ratio: 0.86, 95% CI 0.80–0.93; *P* < 0.001). Fractional flow reserve-guided management reduced MACE (*Figure 3*) and angina. This analysis affirms the prognostic importance of the FFR value (as opposed to an FFR binary cut-off value) and indicates that patients who have a low-normal FFR value, i.e. 0.81–0.85, have a higher likelihood of future adverse cardiac events compared with patients with a near-normal FFR value, i.e. 0.96–1.0. Although evidence from randomized trials is lacking, a pragmatic clinical approach would be to prescribe more intensive preventative therapy in patients with lower FFR values, including after PCI.^80,81^

## Fractional flow reserve in stable coronary disease: results from multicentre clinical studies

**Figure 4:**
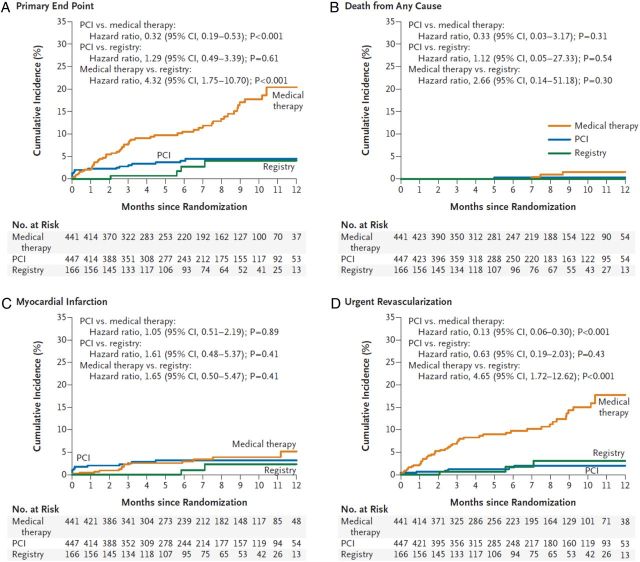
FAME-2 long-term follow-up. Cumulative incidence of the primary endpoint (death, myocardial infarction, or urgent revascularization) and its components.^19^

The FAME-2 trial enrolled 1220 patients with stable CAD.^18^ Of these, 888 patients with at least one functionally significant stenosis (FFR ≤ 0.80) in whom PCI was intended were randomized to either PCI with optimal medical therapy (OMT) or OMT alone. Drug-eluting stents were used almost exclusively. The study was stopped prematurely by the Data Safety Monitoring Board due to a statistically significant reduction in hospital re-admission for urgent revascularization in the PCI group. Clinical outcomes in the PCI patients were similar to those in the registry patients who had no ischaemia in the first place (FFR > 0.80) (*Figure 4*). The FAME-2 design involved unblinded treatment assignment thus participants in the OMT group and their clinicians were aware that PCI had been cancelled by protocol. The definition of urgent coronary revascularization for the primary outcome required both an urgent unplanned hospital admission with persistent or increasing symptoms (with or without ECG changes or elevated biomarker levels) and that the revascularization be performed within 24 h of admission. Cardiologists blinded to the treatment group assignment adjudicated this outcome. The 2-year primary outcome results reaffirmed the initial results.^19^

In patients with moderate–severe ischaemia revealed by non-invasive testing, the impact of invasive management with OMT vs. medical therapy alone on cardiovascular death and non-fatal MI is currently being assessed in the International Study of Comparative Health Effectiveness With Medical and Invasive Approaches (ISCHEMIA) trial (sample size, *n* = 8000).^85^

In summary, in stable CAD, the evidence-base supports revascularization of lesions with an FFR of ≤0.80 whereas CAD associated with an FFR of >0.80 can be managed medically.

## Fractional flow reserve in acute coronary syndromes

The diagnostic validity of FFR is less certain in ACS patients partly because of concerns that the response to pharmacological vasodilatation may be reduced due to culprit artery microvascular obstruction leading to false-negative FFR values. Accordingly, FFR is not valid in the culprit artery of STEMI patients.^86^ However, FFR may be useful in NSTEMI patients since culprit (and non-culprit) antegrade flow is usually preserved.

**Figure 5:**
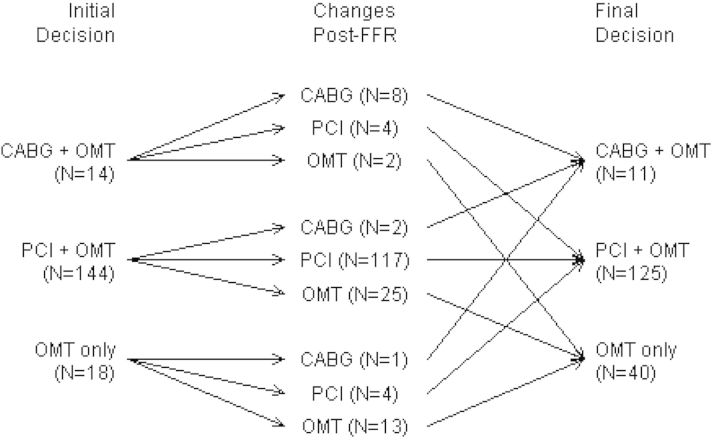
Impact of fractional flow reserve disclosure on treatment decisions based on standard angiography-alone in the FAMOUS-NSTEMI clinical trial.^87^

The FAMOUS-NSTEMI trial (NCT01764334) was the first multicentre, randomized trial of routine FFR-guided management vs. standard invasive management in ACS patients.^87,88^ Three hundred and fifty medically stabilized patients with an NSTEMI were randomized and an initial treatment decision was made following the coronary angiogram and before FFR measurement. Where feasible, FFR was performed in each artery containing at least one lesion of ≥30% diameter stenosis by visual estimation but in the patients randomized to angiographic guidance, the FFR results remained blinded. The primary outcome was the between-group difference in the proportion of patients allocated to medical management. A higher proportion of patients were treated with medical therapy only in the FFR group compared with the angiography-guided group (22.7 vs. 13.2%; *P* = 0.022) (*Figure 5*). In other words, the use of FFR reduced revascularization. As in FAME,^17^ there was marked discordance between the visual assessment of stenosis severity and functional significance defined by FFR.

Of 350 patients (*n* = 706 lesions), an FFR result was obtained in 100% of the participants and only two coronary dissections occurred due to the pressure wire, indicating routine FFR measurement was feasible and safe. There were no adverse events relating to intravenous adenosine. There was no difference in MACE between the groups and other health and economic outcomes were similar.

FAMOUS-NSTEMI^87,88^ differed by design from FAME^17^ in that it only enrolled NSTEMI patients, all treatment options (medical therapy, PCI, and CABG) were possible, FFR was recorded but not disclosed in the angiography-guided group, and the stenosis cut-off value for enrolment in FAMOUS was ≥30% whereas in FAME it was ≥50%. The purpose of adopting this wider range of stenosis severities in FAMOUS-NSTEMI was to provide data on the relationship between ‘mild’ lesions and FFR. A 3.0 Tesla stress perfusion magnetic resonance imaging sub-study in FAMOUS has recently provided evidence that FFR retains diagnostic validity in medically stabilized NSTEMI patients.^89^ A large trial of FFR-guided management in NSTEMI patients that is designed and powered to assess health and economic outcomes now seems warranted.

## Recent developments with fractional flow reserve in the clinical guidelines for stable coronary artery disease

The current guidelines for stable CAD^3^ and myocardial revascularization^4^ reaffirm the diagnostic value of FFR and that FFR-guided PCI with medical therapy is evidence-based to decrease the need for urgent revascularization compared with OMT alone. The guidelines state that deferral of PCI or CABG based on an FFR >0.80 appears safe. The adoption of FFR has a Class I (Level of Evidence A) recommendation when prior evidence of ischaemia is not available. Fractional flow reserve-guided PCI has a Class IIa (Level of Evidence B) recommendation in patients with multi-vessel coronary disease. The guidelines for stable CAD^3^ recommend consideration of direct referral of patients with severe angina or a high pre-test probability of CAD (>85% likelihood) for early invasive coronary angiography, and since information on inducible ischaemia may then be lacking treatment decisions in the catheter laboratory should be informed by measurement of FFR where appropriate (Class I recommendation, Level of Evidence C). Coronary angiography with FFR should also be considered for risk stratification in patients with an inconclusive diagnosis on non-invasive testing or when conflicting results arise from different test modalities (Class IIa, Level of Evidence C). Revascularization of angiographically intermediate lesions without ischaemia or without an FFR < 0.80 is not recommended (Class III recommendation, Level of Evidence B). The guidelines also mention that non-invasive FFR requires further validation before its clinical use may be justified.^3,4^

These guidelines are evidence-based recommendations, but they are not presented as binding requirements. The FFR threshold of 0.80 represents the upper limit of a transition zone for flow-limiting coronary disease, and as mentioned above, patient-specific factors influence the FFR value. Good clinical practice should take into account all relevant information when making a revascularization decision.

## Recent developments on fractional flow reserve in the guidelines for acute coronary syndromes

In the guidelines for the management of patients presenting without persistent ST-segment elevation,^90^ FFR is described as useful in patients with intermediate lesions >5 days after the index event. Fractional flow reserve may be helpful to decide upon the treatment strategy, and in patients with multi-vessel disease, FFR may help to assess non-culprit lesions as part of a sequential management approach involving the ‘Heart Team’. The STEMI guidelines^91^ state that FFR may be used to assess non-infarct arteries as part of a staged revascularization approach planned for days or weeks after the initial primary PCI.

### Current trials in stable coronary artery disease and acute coronary syndrome and future prospects

The current trials of FFR-guided strategies are highlighted in Supplementary material online, *File S1*. The potential clinical utility of an FFR-guided management in STEMI patients with multi-vessel CAD is being studied in DANAMI-3-PRIMULTI,^92^ COMPARE-ACUTE,^93^ and COMPLETE.^94^

## Conclusions and future horizons

As the FFR evidence-base evolves, so will the rationale for functional testing of CAD severity. An assessment of published studies using quality criteria (Supplementary material online, *File S1*) indicates that retrospective analyses in selected patient cohorts involving comparisons of diagnostic indices without masking (blinding) and/or independent analysis are common. The quality of diagnostic studies in the future must improve.

Emerging developments with diagnostic coronary guidewires include a diagnostic rapid exchange FFR microcatheter (ACIST NAVVUS™), and solid-state pressure wires with optical technology with the potential to overcome pressure drift (e.g. OPSENS™; POLARIS™, Boston Scientific), and potentially measurement of absolute coronary flow may become possible.

## Supplementary material

Supplementary material is available at *European Heart Journal* online.

## Authors’ contributions

All of the authors contributed to the literature review, manuscript drafts, and reviewed and approved the final manuscript.

## Funding

D.C. is supported by a British Heart Foundation (BHF) Clinical Research Training Fellowship (FS/14/15/30661). B.H. is supported by a BHF project grant (PG/14/97/31263). Funding to pay the Open Access publication charges for this article was provided by the University of Glasgow.

**Conflict of interest:** The University of Glasgow holds contracts with St Jude Medical for consultancy and research conducted by C.B. K.G.O. has received consultant and speaker fees from St. Jude Medical and Volcano Corporation which manufacture pressure wires. None of the other authors have any potential conflicts of interest.
